# Complete Genome Sequence of Mycobacterium marinum ATCC 927^T^, Obtained Using Nanopore and Illumina Sequencing Technologies

**DOI:** 10.1128/genomeA.00397-18

**Published:** 2018-05-17

**Authors:** Mitsunori Yoshida, Hanako Fukano, Yuji Miyamoto, Keigo Shibayama, Masato Suzuki, Yoshihiko Hoshino

**Affiliations:** a Department of Mycobacteriology, Leprosy Research Center, National Institute of Infectious Diseases, Tokyo, Japan; b Department of Bacteriology II, National Institute of Infectious Diseases, Tokyo, Japan; c Antimicrobial Resistance Research Center, National Institute of Infectious Diseases, Tokyo, Japan

## Abstract

Mycobacterium marinum is a slowly growing, broad-host-range mycobacterial species. Here, we report the complete genome sequence of a Mycobacterium marinum type strain that was isolated from tubercles of diseased fish. This sequence will provide essential information for future taxonomic and comparative genome studies of its relatives.

## GENOME ANNOUNCEMENT

Mycobacterium marinum is a major nontuberculosis mycobacterium, which was first isolated from diseased fish (1) and later shown to be a human pathogen (2). The pathological features of the granuloma are similar to those caused by M. tuberculosis, and the two share many orthologous genes, which is why M. marinum has been used as a surrogate model for tuberculosis (3–6). Another important aspect is that M. marinum is a close relative of M. ulcerans, which produces a macrolide toxin called mycolactone and results in Buruli ulcer (7, 8). Genomic studies indicated that M. ulcerans was diverged from a M. marinum progenitor (9–11). In addition, M. marinum-related mycolactone-producing mycobacteria (MPMs) were also isolated from frogs and fish (12–15). Hence, detailed genomic information of M. marinum will be helpful for understanding evolutionary pathways of these MPMs. Here, we present the complete genome sequence of the first isolate of M. marinum, registered as ATCC 927^T^.

The strain was grown in Middlebrook 7H9 medium, and DNA was extracted by a phenol-chloroform method. Sequence reads (100,513 reads) were obtained with the MinION nanopore sequencer (Oxford Nanopore Technologies, Oxford, UK). Genomic DNA sequencing was performed with the Nanopore SQK-RAD003 rapid sequencing kit in accordance with the manufacturer’s protocol. The library was loaded on a SpotON Mk I (R9.4) flow cell and sequenced using MinKNOW version 1.7.14, and raw sequence data (FAST5 format) were base-called using Albacore Sequencing Pipeline version 2.0.2 software. The reads were *de novo* assembled into two contigs (6,456,544 bp and 173,286 bp) with Canu version 1.5 (15, 16, 17), and the assembled genome was circularized by manually trimming the repeated sequences. Illumina paired-end (2 × 150-bp) reads (266,781,451 reads) were obtained with the MiniSeq system (Illumina, San Diego, CA, USA) and mapped to the assembly using the Burrows-Wheeler aligner (15, 17, 18) for sequence and assembly error correction with Pilon (15, 17, 19). The resulting sequences (chromosome and one plasmid) were annotated using DFAST-core (20). Orthologous gene clusters were identified using Cd-hit (21). Average nucleotide identity (ANI) was calculated by JSpeciesWS (15, 17, 22).

The chromosome of M. marinum ATCC 927 is 6,451,936 bp (65.7% G+C content). The average nucleotide identities to two reported genomes of M. marinum were 98.2% (strain M) and 99.48% (strain E11). The number of predicted coding sequences (CDSs) in the genome (*n* = 5,906) was more than the number of CDSs for strain M (*n* = 5,593) and strain E11 (*n* = 5,383). The numbers of rRNA operons (*n* = 6) and tRNAs (*n* = 51) were equivalent to those of strain E11 but more than those of strain M. We also found a phage-like plasmid (127,216 bp, named pMMRN), whose size is different from that of the plasmid pRAW in strain E11 (114,229 bp) and the plasmid pMM23 (23,317 bp) in strain M. Plasmid pMMRN contains 123 CDSs, whereas 97 and 29 CDSs were present in pRAW and pMM23, respectively. We identified 4,330 orthologous gene clusters among the three strains, whereas 1,223, 714, and 287 gene clusters were specific to ATCC 927, M, and E11, respectively. The complete genome sequence of M. marinum ATCC 927^T^ comprises essential data for future taxonomic and comparative genome studies.

### Accession number(s).

The chromosome and plasmid sequences reported here were deposited in DDBJ/ENA/GenBank under the accession no. AP018496 and AP018497, respectively.
